# Emerging Roles of Circular RNA in Macrophage Activation and Inflammatory Lung Responses

**DOI:** 10.3390/cells13171407

**Published:** 2024-08-23

**Authors:** Chang Jun Son, Jonathan M. Carnino, Heedoo Lee, Yang Jin

**Affiliations:** 1 Division of Pulmonary and Critical Care Medicine, Department of Medicine, Boston University, Boston, MA 02118, USA; cjson@bu.edu (C.J.S.); jcarnino@bu.edu (J.M.C.); leehd@changwon.ac.kr (H.L.); 2 Department of Biology and Chemistry, Changwon National University, Changwon 51140, Republic of Korea

**Keywords:** macrophage activation, inflammation, bacterial infection, circular RNA, endotoxin, extracellular vesicles (EVs), LPS, broncho alveolar lavage fluid (BALF)

## Abstract

Circular RNA (circRNA) is a type of single-stranded RNA that forms a covalently closed continuous loop, unlike linear RNA. The expression of circRNAs in mammals is often conserved across species and shows tissue and cell specificity. Some circRNA serve as gene regulators. However, the biological function of most circRNAs is unclear. CircRNA does not have 5′ or 3′ ends. The unique structure of circRNAs provides them with a much longer half-life and more resistance to RNase R than linear RNAs. Inflammatory lung responses occur in the pathogenesis and recovery of many lung diseases. Macrophages form the first line of host defense/innate immune responses and initiate/mediate lung inflammation. For example, in bacterial pneumonia, upon pro-inflammatory activation, they release early response cytokines/chemokines that recruit neutrophils, macrophages, and lymphocytes to sites of infection and clear pathogens. The functional effects and mechanisms by which circRNAs exert physiological or pathological roles in macrophage activation and lung inflammation remain poorly understood. In this article, we will review the current understanding and progress of circRNA biogenesis, regulation, secretion, and degradation. Furthermore, we will review the current reports on the role of circRNAs in macrophage activation and polarization, as well as in the process of inflammatory lung responses.

## 1. Introduction

Non-coding RNAs are emerging targets for developing novel therapeutic and diagnostic strategies. Among all the types of non-coding RNAs, unlike long non-coding RNAs (lncRNAs) and microRNAs (miRNAs), circular RNAs (circRNAs) carry unique features that have attracted emerging attention recently. Circular RNAs were initially reported in 1976 as RNA viruses [1]. Several years later, the circular form of RNA was identified under electron microscopy in the cytoplasm of eukaryotic cells [2] and mitochondria of yeast [3]. Initially, most circRNAs were thought to be generated by mis-splicing and had no meaningful biological functions [4]. With the development of deep RNA sequencing and bioinformatics, circRNAs are found widely expressed in different species including mammalian cells [5,6,7]. Recently, emerging evidence has shown that circRNAs carry an essential role in various physiological and pathological processes. The involvement of circRNAs in human diseases has been reported, including but not limited to cancer [8,9], cardiovascular disease [10], and neuronal diseases [11]. In the setting of host defense, innate immunity, and inflammatory responses in response to noxious stimuli, circRNAs have been reported to exert antiviral effects [12]. Furthermore, circRNAs are differentially detected in patients with systemic lupus erythematosus (SLE), suggesting their potential to serve as novel biomarkers [13] in the process of inflammation. Inflammatory responses are common features in the lungs in the process of host defense against noxious stimuli, including both infectious and sterile stimuli, such as bacterial, and viral infections, acid inhalational injury, and hyperoxic injury [14,15]. Macrophages including alveolar macrophages (AMs) are the main immune cell population in the lungs and alveolar sacs. They are responsible for maintaining homeostasis and serve as the first line of defense against external insults [16,17]. In this review, we will discuss the current updates on the potential role of circular RNAs in lung inflammation and macrophages.

## 2. Circular RNA Characteristics, Biogenesis, Degradation, and Removal from Intracellular Compartments

Circular RNAs (circRNAs) represent a distinct category of noncoding RNAs characterized by their covalently closed loop structure. This design provides them with a notable resistance to enzymatic breakdown because they do not have the 5′ caps and 3′ tails that are usually targeted by exonucleases. Consequently, circRNAs have at least a 2.5-fold longer half-life than linear RNAs, enhancing their ability to fulfill various roles, such as serving as biomarkers and therapeutic targets for human diseases. [18,19,20,21] The sequences of circRNAs are conservative to a certain extent [21]. Most circRNAs are located in the cytoplasm and some are in the nucleus such as the exon-intro circRNAs (EIcirRNAs) [22,23]. Most circRNAs belong to ncRNAs and are produced by exons [24] despite that certain single gene loci can be cycled into multiple circRNAs and translated into peptides/proteins [24].

**Figure 1:**
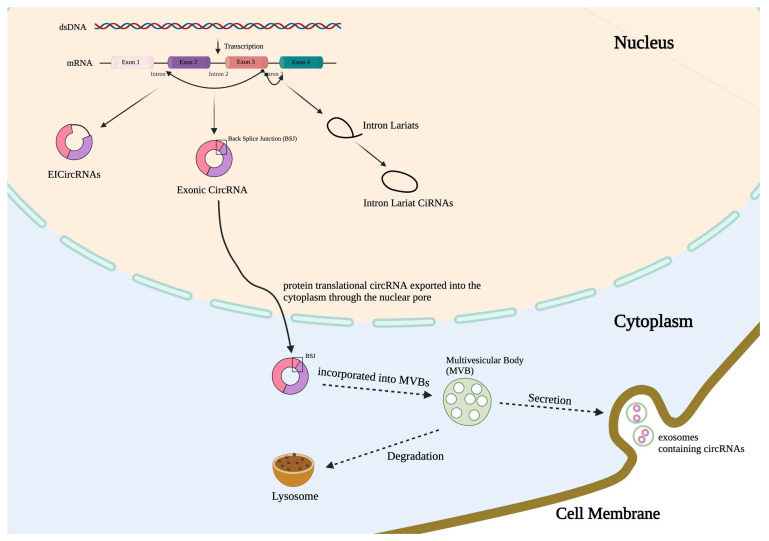
circRNA formation, transportation, and secretion. CircRNAs can be generated by back splicing in four different manners. Here, three common categories of circRNAs are illustrated: intronic circRNAs (ciRNAs); exonic circRNAs, which contain two or more exons or their partial sequences; and exon-intron circRNAs (EIciRNAs), which retain introns between exons. The fourth type of circRNAs refers to the tRNA intronic circular RNAs spliced from the endonuclease complex. CiRNAs are mainly located in the nucleus; exonic circRNAs are often distributed in the cytoplasm and nucleus; and EIciRNAs are located mainly in the nucleus, like ciRNAs. Cytoplasmic circRNAs can be enwrapped into the MVBs and, subsequently, transported to autophagosomes and lysosomes, and/or merged with cytoplasmic membrane and, subsequently, secreted into the extracellular spaces.

CircRNA biogenesis is a specific process unique to each cell type. Most circRNAs are produced utilizing a spliceosome-dependent process known as back-splicing. During this process, a splice donor site downstream connects to an upstream splice acceptor site, crafting a circRNA molecule devoid of the linear RNA’s conventional 5′ cap and 3′ poly-A tail. This circular formation is facilitated by RNA-binding proteins and the intron sequences flanking the exons that will circularize, promoting the close alignment necessary for circRNA creation [25,26,27,28,29,30]. As shown in Schema 1, various types of circRNA formation have been identified. Besides the most recognized spliceosome-mediated back-splicing, other types of circRNAs exist, such as intron-derived nuclear circRNAs. Unfortunately, RNA-seq reads that span back splicing junctions often do not reveal the internal structure of the circular RNA. The most formed circRNAs are produced by exons sharing the same sequences in the cognate linear RNA. CircRNA and its host linear RNA are normally coexpressed. Therefore, the back-splice junction (BSJ) is the only sequence that allows unambiguous identification of the circular form [25,26,27,28,29,30]. To date, circRNA biogenesis is not completely understood. Under the assistance of specific RNA-binding proteins (RBPs), base pairing between inverted repeats in the flanking introns potentially induces back-splicing (Figure 1) [25,26,27]. Based on their sequences, circRNAs are divided into four classes. In addition to the intronic circRNAs (ciRNAs) mentioned above, exonic circRNAs contain two or more exons or their partial sequences. The third type of circRNA refers to the exon-intron circRNAs (EIciRNAs), which retain introns between exons. The fourth type of circRNAs has recently been reported, referring to the tRNA intronic circular RNAs, which are spliced from the endonuclease complex. The differential mechanisms involved in the biogenesis of circRNAs dictate the specific locations of these circRNAs. For example, ciRNAs are mainly located in the nucleus [28]; exonic circRNAs are often distributed in the cytoplasm and nucleus [29]; and EIciRNAs are located mainly in the nucleus, like ciRNAs [30].

As mentioned above, due to the circular form and the absence of a free 5′ cap and 3′ poly A ends, circRNAs are more resistant to exonucleases [5,19], resulting in the accumulation of circRNAs intracellularly. In certain scenarios, the intracellular level of circRNAs exceeds their counterpart linear form RNAs. This raises important questions, e.g., what are the consequences of accumulated circRNAs in the cells? Are cells capable of removing the unwanted circRNAs? Are any human diseases associated with the accumulation of circRNAs? [5,19,31]. Can the circRNAs potentially be diagnostic markers or, more importantly, therapeutic targets?

Although we do not have all the answers to the above questions, a partial understanding of the fates of circRNAs has revealed that cells remove intracellular circRNAs via active secretion. BSJ-containing circRNAs are exported to the cytoplasm. Numerous reports have further demonstrated the presence of abundant circRNAs in extracellular vesicles (EVs) and exosomes. The higher amount of circRNAs were enriched in exosomes compared to the producer cells [32,33], suggesting that EV (exosome)-mediated secretion is one of the mechanisms by which cells remove the accumulated intracellular circRNAs.

There are several hypotheses regarding the encapsulation of circRNAs into EVs. In 2015, circRNAs were first reported to be stably enriched in exosomes [23]. The abundance of circRNAs in exosomes is increased by at least 2- to 6-fold compared with their intracellular levels, suggesting that circRNAs are transported into EVs via an active process. More than 1000 different circRNA candidates have been identified in human serum exosomes [34]. One of the studies has further shown that KRAS (Ki-ras2 Kirsten rat sarcoma viral oncogene homolog) mutations lead to a differential abundance of each specific exosomal-cargo circRNA. Ref. [35], suggesting that circRNAs are selectively packaged into EVs. Interestingly, circRNAs are more pronounced in exosomes, the smaller size EVs, which usually have a different mechanism of generation compared with larger size EVs, which usually are generated via direct plasma membrane budding. EVs are a general term describing lipid bilayer vesicles that are released from cells ranging from 20 to 5000 nm [36]. Based on the current guidelines issued by the International Society of Extracellular Vesicles (ISEV) in 2024 [36], small EVs formerly called exosomes refer to the EVs with a diameter of ~ 30–200 nm that are produced by the fusion of multivesicular bodies (MVBs) with the cell plasma membrane [37]. The molecular mechanisms of exosome biogenesis remain incompletely explored. Early endosomes, the intraluminal vesicles (ILVs), and MVBs are all involved in exosome generation and dynamically communicate with other organelles, such as the trans-Golgi network (TGN) and the endoplasmic reticulum (ER), mitochondrion, or phagosome [38,39,40,41]. MVBs eventually fuse with lysosomes to be degraded or fuse with the plasma membrane to generate exosomes (Schema 1). Larger EVs including formerly called microvesicles (MVs) are often generated by directly budding from the cell membrane [36]. Based on the different mechanisms of EV biogenesis, it is not surprising to us that exosomes consist of the majority of circRNA cargos, given that exosomes experience a long journey from ILV formation along with various cargo incorporations to the fusion of MVBs with the cell membrane. CircRNA-containing mature MVBs can fuse with lysosome or autophagosome to be degraded. That said, circRNAs are much more resistant to nuclease-mediated degradation. It remains unclear what percentage of the MVB-transported circRNAs are degraded in the lysosomes and what percentage of the circRNAs are recycled into the intracellular vesicle systems or secreted via exosomes.

Scattered reports involving circular RNA degradation show that specific endonucleases participate in circRNA degradation. RNase H1 cleaves the R-loops formed between the circular intronic RNAs and the DNA at their expression sites, such as *ci-ankrd52*. Therefore, *ci-ankrd52* can be degraded via RNase H1 [42]. The circRNAs with high GC content are more likely to form R-loops, and cells can recruit RNase H1 to degrade circRNAs in such R-loops [42]. RNase L has been reported to restrict circPTPN22 [43]. Upon inflammation or virus infection, circRNAs have been reported globally degraded by activated RNase L [44]. The second key components involved in circRNA degradation are RNA binding proteins. Argonaute protein-2 (Ago2) recognizes and cleaves the complex formed by miRNA-1224 and pre-circRNA-Filip1l, leading to a decreased mature circRNA-Filip1l in the spinal nucleus [45]. The miRNA-671-circRNA-CDR1 complex is cleaved by Ago2 and degraded afterward [46]. GW182 deletion results in elevated circdati and circlaccase2 levels [47]. GW182 may regulate circRNA degradations in an Ago-slicer- or P-body-independent manner via the ABD and/or the UBA [47]. CircRNAs can also be degraded via structure-mediated RNA decay (SRD). SRD is a specific mechanism by which the highly folded and structured RNA is degraded. RNA-binding proteins, up-frameshift protein 1 (UPF1), and Ras-Gap-SH3 domain-binding protein 1 (G3BP1) facilitate the RNAs to form the highly structured RNA [48]. Recent studies suggest that UPF1 and G3BP1 bind to highly structured base-pair regions of circRNAs and promote circRNA degradation [48].

Thus far, we do not have a complete understanding of the selection of specific circRNAs that are transported from the nucleus to cytoplasm and/or secreted from the cells to exosomes. However, the lengths of mature circRNAs potentially dictate nuclear exportation. Two closely related RNA helicases, URH49 and UAP56, control the transportation of short and long circRNAs from the nucleus, respectively [49]. Next, the post-transcriptional modifications (PTMs) play a role in the nuclear export and/or secretion of the circRNAs. N6-methyladenosine (m^6^A) modification regulates the circRNA biogenesis, the nuclear export of circRNAs, and the degradation of circRNAs [50,51,52,53,54,55]; YTHDC1, an m^6^A reader, promotes the nuclear export of m^6^A-modified circNSUN2 [50]. EIF4A3, another m^6^A reader, increases the nuclear export of circPRKCI [51,52]. The deletion of Exportin 4 (XPO4) results in the nuclear accumulation of exonic circRNAs, subsequently leading to the formation of harmful R-loops [53].

There are several hypothetic mechanisms of the circRNAs sorting into exosomes. First, the size of the circRNAs potentially dictates their encapsulation into the exosomes. The average size of the circRNAs that were not secreted from cells was 459 nucleotides (nts), while the average size of the circRNAs released by exosomes was 435 nts [54]. Next, RNA binding proteins (RBPs), such as hnRNPs, have been shown to mediate microRNA(miRNA) encapsulations into the exosomes [55]. MiRNAs and other long-non-coding RNAs potentially competitively regulate circRNA sorting into exosomes. One example is that the deletion of lncRNA UCA1 in serum exosomes leads to an elevated circHIPK3 expression, indicating a competitive mechanism of lncRNA UCA1 and exosomal circHIPK3 [56]. The sponging effects of miR-7 against circCDR1 raised intracellular levels of miR-7, diminished the circCDR1 in exosomes, and up-regulated the circCDR1 level in the cells [23]. That said, the regulations and mechanisms involved in the exosomal secretion of circRNAs remain largely unexplored.

## 3. Circular RNA and Its Potential Roles in Macrophage Activation and Inflammatory Lung Responses

CircRNA has been reported to regulate a variety of human diseases including but not limited to aging-related diseases, cancer, cardiovascular diseases, diabetes, osteoarthritis, stress, and viral diseases [57,58,59,60,61,62,63,64]. CircRNAs have also been reported to be involved in pulmonary fibrosis, acute respiratory distress syndrome (ARDS), cystic fibrosis, pulmonary hypertension, pulmonary tuberculosis, asthma, and silicosis [65,66,67,68,69,70,71,72,73,74]. Inflammatory lung responses often play an important role in the pathogenesis of these pulmonary disorders. Macrophages are the first-line cells in host defense/innate immunity and regulate the initiation, maintenance, and resolution of the inflammatory response. Here, we will review the current understanding of the role of circRNAs in inflammatory lung responses and macrophage activations.

## 4. CircRNAs and Macrophage Activation, Differentiation, and Polarization

CircRNAs are reported to regulate macrophage polarization and activation in response to LPS. CircRNAs can also inhibit macrophage biogenesis [75,76,77]. In one study, approximately 2000 circular RNAs were altered after TLR4 stimulation. In response to LPS stimulation, circRNA circRasGEF1b is stably expressed and required for NF-kB and macrophage activations [75,76,77]. Furthermore, Gonzalez et al. demonstrated the effects of circRNAs on macrophage polarization using cirRNA cdr1as as an example [78]. In this report, the authors performed circRNA microarray analyses in bone marrow-derived macrophages (BMDM) which were differentiated into the pro-and anti-inflammatory phenotypes. Using circ-Cdr1as as an example, the authors investigated the macrophage polarizations using the “gain or loss of function” approaches by deletion or overexpression circ-Cdr1as in BMDMs. The author showed that Cdr1as is one of the most downregulated circRNAs in pro-inflammatory macrophages and robustly upregulated in anti-inflammatory macrophages. Circ-Cdr1as potentially promotes anti-inflammatory markers and steers the cell toward M2 polarization. Circ17725 has also been demonstrated to be downregulated in peripheral blood mononuclear cells (PBMCs) obtained from RA patients. Circ17725 is found to interact with miR-4668-5p-FAM46C. Functionally, circ17725 inhibits the proliferation and enhances the apoptosis of macrophages. Additionally, overexpression of circ17725 decreases the expression of TNF-α, IL-1β, and MMP-9 in Raw264.7 macrophages, suggesting that it promotes macrophage polarization towards M2 by targeting miR-4668-5p/FAM46C as a miRNA sponge. CircANKRD36 sponges miR-330, resulting in increased ROCK1 in LPS-treated Raw264.7 macrophages [79].

One recent report analyzed the circRNA expression in polarized human macrophages. The authors demonstrated that 9720 circular RNAs are detected using RNA sequencing in human THP-1 macrophages. A total of 71 up-regulated circRNAs and 69 down-regulated circRNAs are detected in the M1-geared THP-1 cells, confirmed by Quantitative real-time PCR (qRT-PCR) [80]. Using circRNA-RNF19B (circRNF19B) as an example, it is significantly up-regulated in M1 macrophages. On the other hand, circRNF19B expression is increased when the M2 phenotype is converted to M1 and vice versa. Additionally, circRNAs are differentially expressed in human macrophages after *Mycobacterium tuberculosis* infection; 32 circRNAs were up-regulated and 110 were down-regulated [81]. The author hypothesized that circ_0043497 and circ_0001204 are potentially effective diagnostic biomarkers for TB. In septic patients, circular RNA ASPH (circASPH) is highly expressed in peripheral blood mononuclear cells. CircASPH levels peaked after 24 h of M1 polarization and after 12 h of M2 polarization in THP1 cells [82]. Deletion of circASPH leads to the downregulation of M1 gene expression and cytokine secretion.

CircRNAs can be secreted into extracellular space via exosomes. Therefore, not only do the endogenous circRNAs play a role in macrophage activation, but the exogenous circRNAs, after entering the target cells, potentially regulate the cellular events of the recipients. Recent reports show that exogenous circRNAs interact with RNA sensors in macrophages [83]. Delivery of exogenous circRNAs potentially stimulates a greater innate immune response than that stimulated by their linear counterparts [83]. The transfection of purified in vitro-produced circRNAs into the recipient cells results in significant innate immune responses against viral infection [83]. Furthermore, Retinoic acid-inducible gene I (RIG-I) has been demonstrated to sense foreign circRNA and co-aggregate in cytoplasmic foci. The introns involved in circRNAs generation play an essential role in the recognition of foreign circRNAs and initiate immune responses. The summary of the recently discovered circRNAs that play a role in regulating macrophage activation, apoptosis, and polarization are listed in Table 1.

## 5. circRNAs in Inflammatory Lung Responses

Although macrophage forms the first line of innate immunity, inflammatory lung responses involve many more cell types and pathways. Therefore, many more studies have reported the potential roles of circRNAs in the initiation, development, and resolution of lung inflammation and, in the end, lung injury. These reports revealed that not only macrophages but also epithelial cells, endothelial cells, dendritic cells, and fibroblasts all potentially are regulated by circRNAs in the process of inflammatory lung responses. For example, the deletion of circ0038467 reduces LPS-induced inflammatory injury [100]. CircHECTD1 inhibits the apoptosis of AECs [101]. CircPALM2 is increased in LPS-caused MLE-12 cell damage [102]. Circ0054633 is over-expressed in LPS-induced rats and murine pulmonary microvascular endothelial cells, through activating the NF-κB pathways [103]. Deletion of circANKRD36 suppressed cell viability and migration and alleviated inflammation of LPS-treated macrophages, probably serving as a sponge for miR-330 and ROCK1 [79]. Circ_0001679 is upregulated in LPS-induced MLE-12 cells and leads to increased apoptosis [104]. Furthermore, LPS induces circTMOD3 in WI-38 lung fibroblasts, and circTMOD3 sponges miR-146b-3p to induce CXCR1 expression [105]. Circ_VMA21 was downregulated in LPS-treated WI-38 cells after pneumonia, and circ_VMA21 sponges miR-409-3p to induce the expression of Kruppel-like transcription factor 4 (KLF4), an inflammatory palliative in sepsis [106].

However, many more studies did not focus on specific cells; rather, they used in vivo studies to demonstrate the overall picture. Circ42341, circ44122, and circ44123 were robustly upregulated, whereas circ010498, circ25030, and circ010498 were significantly downregulated through microchip analysis in total lung tissue from the LPS-mouse lung injury model [107]. CircC3P1 reduces pro-inflammatory cytokine production and cell apoptosis [108]. CircVMA21 decreases oxidative stress, apoptosis, and inflammation in sepsis rats [109]. Circ0001679 and circ0001212 are increased in septic mice and regulate Nprl3 inflammasome expression [110]. CircNCLN sponge and antagonize miR-291a-3p to alleviate LPS-induced acute lung injury [111]. In the plasma of influenza A-induced lung injury patients, circRNAs Slco3a1 and Wdr33 were aberrantly expressed [84]. CircVPS33A and circ_0000455 are highly expressed in a murine asthma model. CircVPS33A sponges miR-192-5p to increase the level of high-mobility group box 1 (HMGB1), a strong pro-inflammatory mediator [112,113] Many current studies in lung inflammation focus on specific lung cells and lung tissue. Our newly published study showed that the profiles of circRNAs in BALF exosomes are also significantly altered in the setting of bacterial infections [114]. The exosome-cargo circRNAs potentially can travel to adjacent and distant target cells and mediate signal exchanges among cells.

## 6. circRNAs Derived from Microbes Participate in the Pathogenesis of Lung Inflammation and Injury

In addition to host cell-derived circRNAs, circRNAs derived from microbes contribute significantly to human diseases, including but not limited to respiratory disorders. CircRNAs can be derived from both DNA and RNA viruses. A repertoire of latent and lytic viral circular RNAs has been well studied and reported in the Epstein Barr virus (EBV) transcriptome [115]. Its oncogenic role in liver disease has been studied. EBV circRNAs have also been detected in patient samples [116]. In brief, the mechanisms through which circRNAs regulate pathogenesis include but are not limited to miRNA sponges [117], protein sponges, regulating parental gene expression, and peptide translating [118,119].

Besides the well-studied circRNAs in liver diseases, virus-derived circRNAs have also been reported in respiratory disorders. For example, circRNAs are reported to be generated from MERS-CoV, SARS-CoV-1, and SARS-CoV-2, the RNA coronaviridae that cause acute lung injury and have caused several endemic/pandemic episodes [120]. In 2022, Yang et al. predicted that 351, 224, and 2764 circRNAs derived from severe acute respiratory syndrome coronavirus 2 (SARS-CoV-2), SARS-CoV, and Middle East respiratory syndrome coronavirus, respectively [121]. They further confirmed that 75 SARS-CoV-2 circRNAs are potentially derived from SARS-CoV-2. Computational analysis indicated that SARS-CoV-2–circRNAs–miRNAs potentially promote the host genes involved in the response to the virus and muscular skeletal development [122]. Further biomedical informatic analysis suggests that the viral circular RNAs target the host genes regulating, “cytokine receptor binding” and “growth factor activity”. For example, SARS-CoV-2 circRNA 29122|28295 contains the binding site of hsa-miR-3194-5p, and functions as a sponge for host cellular miRNAs, subsequently manipulating the downstream host gene GPR*115*.

In addition to virus-derived circRNAs, circular RNAs derived from bacteria have been reported lately. He et al. demonstrated that a regulatory noncoding RNA (DucS) carries both linear and circular conformation derived from *Bacillus altitudinis* [123]. The circular form of DucS is likely regulated by the 3′ end sequences of the linear forms. Interestingly, the linear forms of DucS enhance the tolerance of oxidative stress by *B. altitudinis* via increased translation of *htrA* stress-responsive gene. The formation of circular DucS potentially decreases the levels of the regulatory linear counterpart, leading to intolerance to H_2_O_2_. Moreover, via bioinformatic analysis, 30 bacterial species have been identified to potentially produce circular forms of RNAs. The authors validated four and seven circular RNAs from *Bacillus paralicheniformis* and *B. altitudinis*, respectively. However, the predicted circular RNAs from *Bacillus subtilis* and *Escherichia coli* were not confirmed, suggesting the circulation of specific RNAs may not be conserved among different bacterial species.

*Trichophyton rubrum* (*T. rubrum*) belongs to human pathogenic filamentous fungi. Cao et al. identified 4254 circRNAs in *T. rubrum* and found 940 differentially expressed circRNAs between the conidial and mycelial stages [124]. Interestingly, they found that unlike the circRNAs discovered in plants and animals, most circRNAs in fungi originated from intergenic regions. Functional analysis suggested that these circRNAs may regulate posttranscriptional processes and protein synthesis. Numerous other reports confirmed that circRNAs exist in the world of fungi that cause both human and plant disorders [125].

## 7. Remaining Questions and Future Directions

One of the most studied mechanisms is circRNA-miRNA sponges. Most of the studies mentioned above have revealed these findings. However, as part of non-coding RNAs, circRNAs are likely to also modify DNA structure and modulate RNA transcription and protein translation. EcircRNAs have been reported to be translated into proteins. Without a 5′cap, circRNAs adopt a cap-independent translation mechanism. They potentially use IRES or MIRES to bind to the initiation factor eIF4G2 complex and anchor the 43S complex for protein translation [85,126]. The next direction regarding the circRNAs and lung inflammation will be to explore the circRNA secretion and reuptake. To date, it remains unclear on which regulators control circRNA secretion, degradation, and uptake. Additionally, most works study the ecircRNAs generated via back-splicing. Intronic circRNAs and EIcircRNAs, as well as tRNA-facilitated circRNAs, require further investigation.

In summary, circRNAs have become a newly targeted non-coding RNA category and potentially have essential roles in the process of inflammatory lung responses and macrophage polarization.

## Figures and Tables

**Table 1:** CircRNAs in Macrophage Activations.

Year	Conclusions/Findings	Citation
2022	circRNF19B increased when M2 converted to M1.	PMID 35368656 [80]
2022	circ-Cdr1as serves as an anti-inflammatory regulator in BMDM.	PMID 36181865 [78]
2022	circCdyl promotes M1 polarization.	PMID 34547461 [18]
2023	circATP9A promotes macrophage M2 polarization	PMID 38049814 [57]
2023	circ17725 Promotes Macrophage Polarization towards M2	PMID 37035757 [84]
2020	circPPM1F modulates M1 macrophage activation	PMID 33042261 [85]
2023	circmSCAR in septic mice is closely related to M1 macrophage polarization.	PMID 36965082 [86]
2018	circZC3H4 RNA/ZC3H4 promotes pulmonary macrophage activation.	PMID 29401612 [87]
2016	circRasGEF1B upregulates LPS-induced ICAM-1 expression in macrophages	PMID 27362560 [77]
2016	circ-ANRIL is involved in the apoptosis of macrophages	PMID 27539542 [75]
2017	circ003780, circ010056, and circ010231 are upregulated and circ003424, circ013630, circ001489 and circ018127 are downregulated in M1.	PMID 28075448 [88]
2019	11 and 126 circRNAs are upregulated and downregulated in lung macrophages in CLP/sepsis mice	PMID 31411002 [89]
2022	CircRNA PLCE1 promotes M2 polarization of tumor-associated macrophages	PMID 35349390 [90]
2023	Circ0075723 inhibits macrophage pyroptosis via miR-155 sponging	PMID 36923408 [91]
2023	*circSOD2*, *circCHSY1*, *circTNFRSF21*, and *circDHTKD1* are differentially expressed in TB-infected macrophages	PMID 38139387 [92]
2023	circASPH upregulates M1 gene expression in macrophage in sepsis.	Western Univ. Thesis [82]
2022	circITGB6- mediated tumor- associated macrophage (TAM) polarization into M2 phenotype	PMID 35277458 [93]
2019	circRNAs (092520, 102610, 004662, or 103124) in PBMCs increase in CD,	PMID 31261517 [94]
2023	circP4HB promoted M2 macrophage phenotype	PMID 37880717 [95]
2021	circHIPK3 sponges miR-192 and miR-561 to promote NLRP3 in macrophages	PMID 34085163 [96]
2020	circRNA-0003528 promoted tuberculosis associated macrophage polarization	PMID 33318319 [97]
2021	Circ0110102 inhibits macrophage activation	PMID 33891564 [98]
2024	circ0000381 promotes microglial/macrophage pyroptosis	PMID 38466640 [99]
